# Prevalence of SARS-CoV-2 virus in saliva, stool, and urine samples of COVID-19 patients in Bihar, India

**DOI:** 10.1099/acmi.0.000693.v4

**Published:** 2024-06-14

**Authors:** Nupur Meghna, Archana Archana, Divendu Bhushan, Abhyuday Kumar, Asim Sarfraz, Bijaya Nanda Naik, Binod Kumar Pati

**Affiliations:** 1 Department of Microbiology, All India Institute of Medical Sciences, Patna, India; 2 Department of General Medicine, All India Institute of Medical Sciences, Patna, India; 3 Department of Anaesthesiology, All India Institute of Medical Sciences, Patna, India; 4 Department of Community and Family Medicine, All India Institute of Medical Sciences, Patna, India

**Keywords:** multiplex RT-PCR, non-respiratory samples, severe acute respiratory syndrome coronavirus 2, transmission route

## Abstract

**Introduction.** The coronavirus illness caused by SARS-CoV-2 can cause multiple organ involvement, with varying degrees of severity. Besides inhalation as a route for transmission, feco-oral has also been proposed. Its transmission to sewage systems is a growing public health issue.

**Objective.** To detect SARS-CoV-2 RNA in non-respiratory samples (saliva, urine, and stool) collected from COVID-19 cases, in Bihar.

**Methods.** This cross-sectional observational study was conducted from January 2021 to March 2022 on human non-respiratory samples. A total of 345 samples including saliva (116), stool (97), and urine (132) were collected from 143 COVID-19 cases. Samples were analysed for SARS-CoV-2 by multiplex RT-PCR targeted against E, ORF 1ab, and RdRp genes.

**Results.** In this study, out of 143 cases, a total of 107 (74.8 %) were positive for SARS-CoV-2 RNA in at least one of the non-respiratory samples.

**Conclusion.** There is a high prevalence of SARS-CoV-2 virus in non-respiratory samples.

­

**Impact Statement**

The non-respiratory samples can serve as a self-collected alternative specimen. Salivary load in asymptomatic carriers can be analysed to establish a sensitivity threshold for future diagnostic rapid tests. This study possesses a potential role in epidemiological data by environmental surveillance (sewage).

## Data Summary

The authors confirm all data or data analysis files provided within the article can be found on Figshare: https://figshare.com/s/b323e539162aa675c11c[1].

## Introduction

The coronavirus illness is caused by Severe Acute Respiratory Syndrome Coronavirus 2 (SARS-CoV-2) which is a zoonotic virus, with a pandemic potential. While the literature suggests human-to-human transmission by the inhalation of respiratory droplets, the possibility of additional transmission pathways like the feco-oral route deserves further research [2].

COVID-19 patients can shed SARS-CoV-2 RNA in various body fluids like saliva, faeces, and urine which suggests the possibility of transmission via non-respiratory routes as well [3]. SARS-CoV-2 RNA excretion via the non-respiratory routes may be a potential source of infection, particularly in rural areas where open defecation and urination are still a rampant practice. As a result, sampling from non-respiratory channels should be done to investigate probable mechanisms of non-respiratory transmission.

In addition to this, the presence of SARS-CoV-2 in human faeces and its transmission to the sewage system is a growing public health issue [4]. As a result, the sewage system might be a potential source of viral outbreaks, community spread, and a future surveillance strategy.

Poor sanitation and insufficient treatment in poorer countries can lead to non-point contamination of surface waters [5]. The non-respiratory samples can serve as a self-collected alternative specimen and help decrease the risk of exposure while collecting respiratory samples by healthcare workers. This study aimed to detect the SARS-CoV-2 RNA in saliva, urine, and stool, besides respiratory specimens.

## Methods

The present study was approved by the Institutional Ethical Committee [Ethics Committee Approval letter no: AIIMS/Pat/IEC/PGTh/Jan20/22]. This cross-sectional observational study was conducted at Virus Research and Diagnostic Laboratory (VRDL), Department of Microbiology, All India Institute of Medical Sciences, Patna between January 2021 and March 2022. The sample size of 132 patients was calculated using OpenEpi software, assuming COVID-19 RNA positivity in various non-respiratory samples with 84.8 % in saliva with a 95 % confidence interval, 7 % absolute precision, and 20 % non-response rate [6]. A total of 143 admitted patients who were real-time PCR SARS-CoV-2 RNA positive in nasopharyngeal or oropharyngeal samples and who gave written informed consent were included in the study. Nasopharyngeal or oropharyngeal real-time PCR SARS-CoV-2 RNA negative cases, non-admitted cases or those who did not give consent were excluded. A total of 345 non-respiratory samples including saliva (116), stool (97), and urine (132) were collected from the admitted 143 COVID-19 cases during the study period. The patient’s demographic data, comorbid conditions, and clinical presentation were recorded. The patient’s respiratory illness was characterized as mild, moderate, or severe based on standard criteria for COVID-19 [7]. The nasopharyngeal or oropharyngeal samples were collected in Viral Transport Media (VTM) [8,10]. The saliva sample was collected by passive drooling technique in a 15 ml centrifuge tube [11]. Urine and stool samples were collected in a sterile universal container. The samples were transported to the laboratory in triple-layered packs under strict aseptic conditions with frozen ice packs (cold chain) [12]. The biological fluids were stored at −20 °C temperature. Standard precautions were followed during sample handling. All the samples were processed inside a Biosafety cabinet type IIA2. The saliva samples were vortexed for 5 min and subjected to RNA extraction. Urine samples were centrifuged twice at 1 200 *g* followed by 12 000 *g* for 10 min and the pellets were collected for the RNA extraction after discarding the supernatant [13]. Two grams of stool was mixed with one gram of sterile glass beads and 5 ml of Phosphate buffered saline (PBS) in a 15 ml Falcon tube [14]. The mixture was vortexed for 20 min while the supernatant was collected after centrifuging at 1 500 *g* for 20 min at 4  °C. The RNA extraction was done with Zybio nucleic acid extraction kit (magnetic bead method) in all three samples. All the reagents were brought to room temperature and mixed well before use. The working solution was prepared by mixing extraction reagent I (500 µl), magnetic bead solution (4 µl), and proteinase K (15 µl). The working solution (500 µl) was transferred to a 1.5 ml centrifuge tube and the lysate was prepared by adding 200 µl of sample to it. It was mixed well for 5 s. The resulting solution was incubated at 55 °C for 4 min in a dry water bath followed by centrifugation for 5 s. Then it was placed on the magnetic separator for a minute and the supernatant was discarded. This was followed by the washing step by adding 600 µl extraction reagent II and mixing for 5 s followed by centrifugation and placing on the magnetic separator for a minute. The supernatant was discarded. Elution was performed by adding 50 µl elution buffer, mixing well for 5 s followed by a short spin. The elute was incubated in a dry water bath at 80 °C for 2 min followed by placing it on the magnetic separator for 1 min. The supernatant was discarded and the residual liquid at the bottom of the tube was removed after 1 min of standing. The centrifuge tube was placed on the magnetic separator and the supernatant was taken out (RNA template). The concentration and purity of nucleic acids (absorbance ratio at 260/280nm) were determined by spectrophotometry using the NanoDrop 1 000 (Thermo Fisher Scientific). Master mix preparation was done according to the manufacturer’s instructions. NIV multiplex single tube SARS-CoV-2 RT-PCR assay (TaqMan fluorogenic probe-based) was used for SARS-CoV-2 detection. One screening gene (E gene) along with two confirmatory genes (ORF 1ab, and RdRp) were utilized for SARS-CoV-2 detection. The PCR protocol was set up in ABI Quant Studio 5 Dx equipment. NIV multiplex single tube SARS-CoV-2 RT-PCR assay consists of reverse transcription, polymerase activation, and amplification steps for 40 cycles were used. The graph was interpreted after observation of the amplification curve and the cut-off CT was 35 as per the kit.

## Statistical analysis

Data was entered in and statistically analysed using SPSS V.21. Continuous variables were expressed as mean (SD) or median (IQR). Categorial variables were expressed as proportions or percentages. Age distribution was compacted into three categories: ≤20 years, 21–59 years, and ≥60 years for analysing prevalence and association. The correlation of CT values of NP/OP samples with the CT value of saliva and stool samples was tested by Pearson correlation test. The association of positivity for SARS-CoV-2 in non-respiratory samples with age, gender, comorbidity, and severity was tested using the Chi-square test and Fisher’s Exact test. A *p*-value<0.05 was considered statistically significant.

## Results

**Fig. 1.**
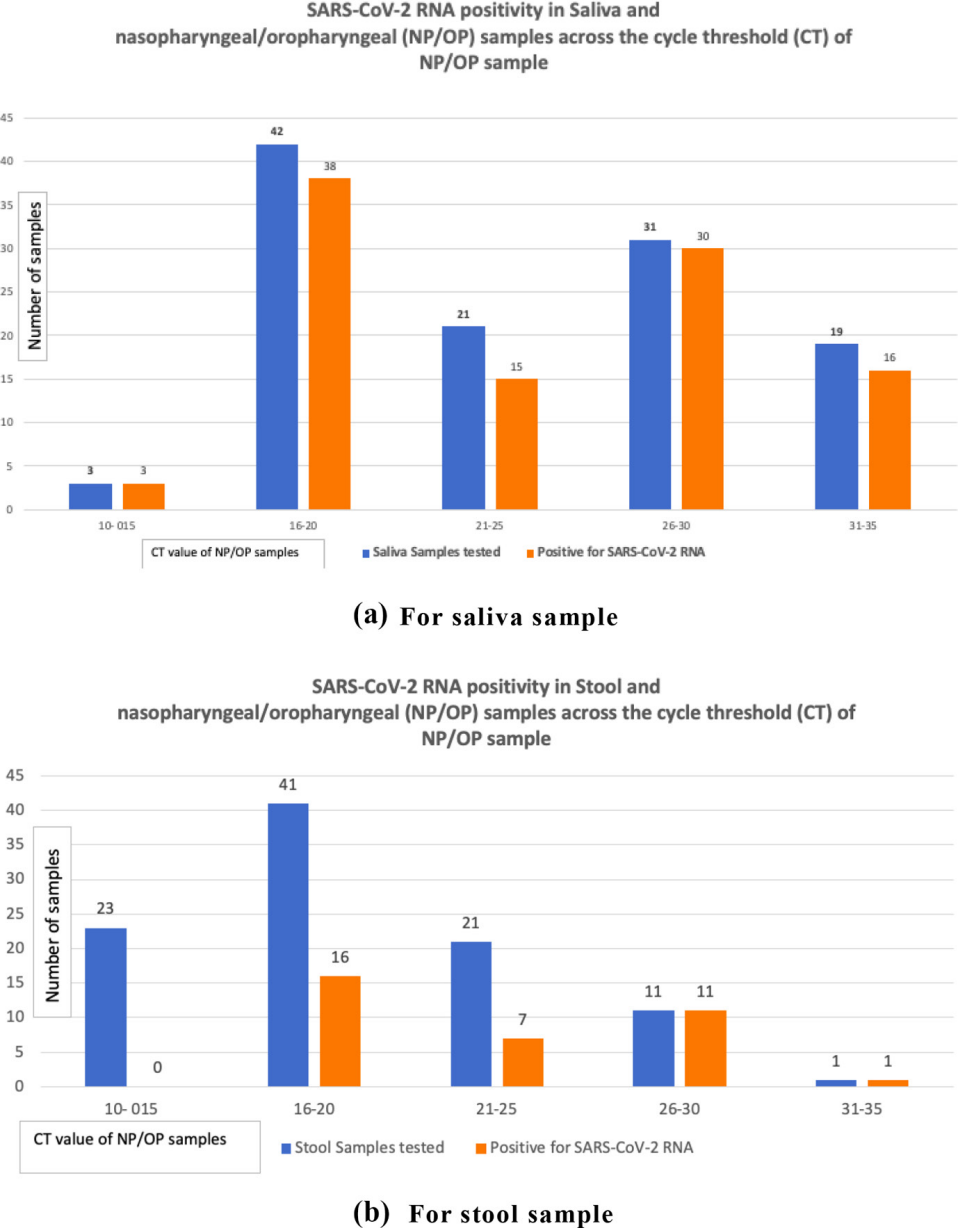
SARS-CoV-2 RNA positivity in non-respiratory and nasopharyngeal/oropharyngeal (NP/OP) samples across the cycle threshold (CT) of NP/OP samples.

**Fig. 2.**
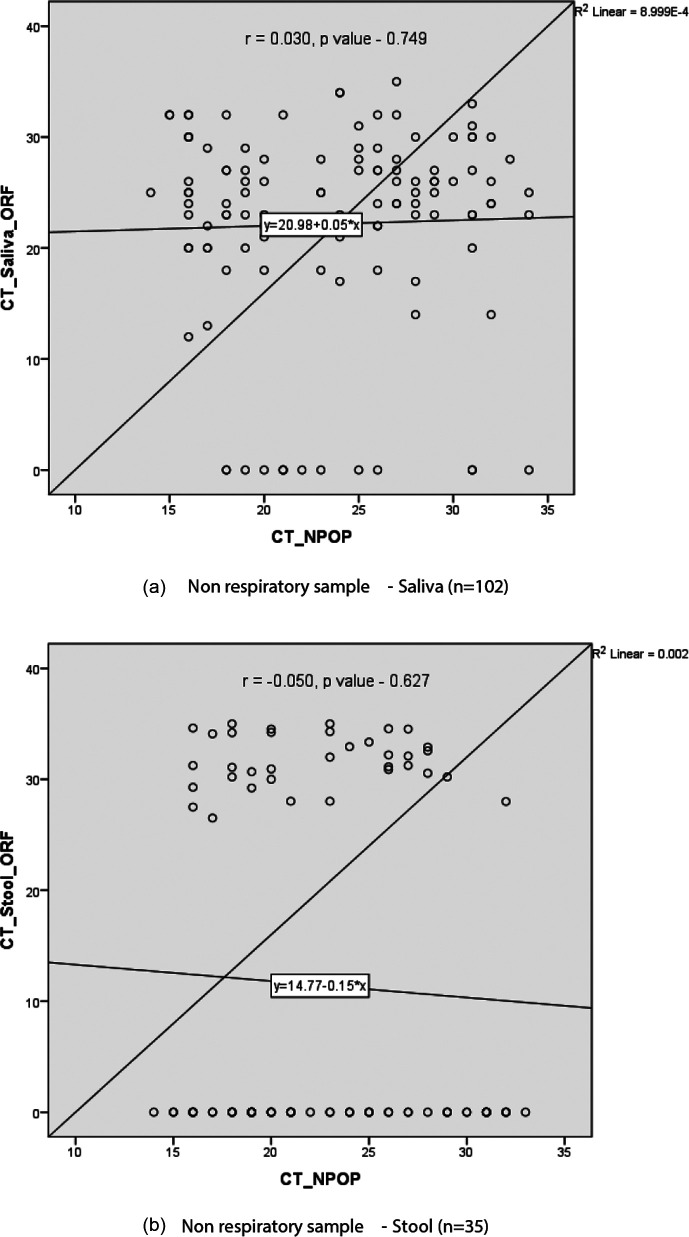
Correlation of the cycle threshold (CT) of SARS-CoV-2 RNA in non-respiratory samples and nasopharyngeal/oropharyngeal (NP/OP) samples.

A total of 143 RT-PCR-positive COVID-19 patients were enrolled in the study between January 2020 and March 2021. A total of 345 non-respiratory samples including saliva 116 (33.6 %), stool 97 (28.1 %), and urine 132 (38.3 %) were obtained and tested for SARS-CoV-2 RNA from these patients. About three-fourths of the patients were male 111 (77.6 %). Most (74 %) of the patients were in the age group of 21 to 59 years with a mean age of 47. About 25 % of patients were ≥60 years of age and only two (1.4 %) patients were of ≤20 years. A total of 96 (67.2 %) cases had one or more comorbidities. Hypertension (51, 33.2 %) was found to be the commonest comorbidity followed by diabetes mellitus (50, 32.5 %) and pulmonary disease (44, 28.5 %). About 55 % (84 patients) had a single comorbidity, 58 (37.6 %) patients had two, 11 (7.2 %) patients had three, and one patient had four comorbidities. The most common presenting complaint was fever in 124 (86.7 %), followed by shortness of breath with cough and chest pain in 103 (72.0 %). The severity of COVID-19 disease was mild in 46 (32.1 %), moderate in 44 (30.8 %), and severe in 53 (37 %) cases [Table 1]. The overall prevalence of the SARS-CoV-2 virus in non-respiratory samples was 39.7 % (95 % C 34.24–44.5 %). The prevalence in saliva and stool samples was 87.93% (95 % CI 82–93.86 %) and 36.08% (95 % CI 26.53–45.64 %) respectively. The SARS-CoV-2 RNA was not detected in any of the urine samples [Table 2]. Out of 143 cases, a total of 107 (74.8 %) were positive for SARS-CoV-2 RNA in at least one of the non-respiratory samples. The SARS-CoV-2 RNA was detected in almost equal proportion in male 86 (77.4 %) and female 21 (65.6 %) patients. An almost equal proportion of non-respiratory samples were positive for SARS-CoV-2 RNA in the age group of 21 to 59 years 77 (74 %) and in ≥60 years 28 (75.6 %). All cases ≤20 years (100 %) were detected with SARS-CoV-2 RNA in non-respiratory samples. The patients with comorbidities had higher SARS-CoV-2 RNA positivity in non-respiratory samples compared to those without a comorbidity (75 [78.1 %] vs 32 [68.1 %]). The highest SARS-CoV-2 RNA positivity in non-respiratory samples was found among moderate COVID-19 patients (38 [86.3 %]) followed by mild (35 [76 %]) and severe (34 [64.1 %]) patients. The difference in the proportions of SARS-CoV-2 RNA in the non-respiratory samples across age, gender, comorbidity, and severity was statistically not significant (*p*-value>0.05) [Table 3]. The SARS-CoV-2 RNA was more prevalent in elderly (≥60 years) than adult (21–59 years) patients in both saliva (71.4 % vs 70.8 %) as well as in stool (34.3 % vs 21.7 %). Similarly, the positivity of SARS-CoV-2 RNA in males was higher than in females in both saliva (73.9 % vs 62.5 %) as well as in stool (28.8 % vs 9.4 %). The positivity of SARS-CoV-2 RNA in patients with a comorbidity was higher than those without comorbidity in both saliva (74 % vs 66 %) and stool (28 % vs 17 %) samples. However, the maximum positivity of SARS-CoV-2 RNA in saliva was observed in patients with moderate as opposed to those with mild (86.4 % vs 69.6 %) disease. On the contrary, the positivity of SARS-CoV-2 RNA in stool was higher in patients with mild than in those with moderate (32.6 % vs 29.5 %) disease. The patients with severe diseases had a relatively low prevalence of SARS-CoV-2 RNA in saliva (60.4 %) as well as in stool (13.2 %) samples [Table 4]. The cycle threshold (CT) value for SARS-CoV-2 RNA detection in saliva was between 10–15 in four (3.9 %), 16–20 in 12 (11.7 %), 21–25 in 37 (36.2 %), 26–30 in 35 (34.3 %) and 31–35 in 14 (13.72 %) cases; while in stool CT value was 26–30 in eight (22.2 %) and 31–35 in the remaining 27 (77.8 %) cases. Across CT values 10–15 of nasopharyngeal/oropharyngeal (NP/OP) samples, almost 100 % of saliva tested positive for SARS-CoV-2 RNA, while none (0 %) of stool tested positive for SARS-CoV-2 RNA. Similarly, 38 (90.4 %) out of 42 saliva and 16 (39 %) out of 41 stools tested positive for SARS-CoV-2 RNA across CT values of 16–20 of the NP/OP sample. Across CT values 21–25 of the NP/OP sample, 15 (71.4 %) out of 21 saliva and seven (33.3 %) out of 21 stools tested positive for SARS-CoV-2 RNA. Across CT values 26–30 of the NP/OP sample, 30 (96.7 %) out of 31 saliva and 11 (100 %) out of 11 stools tested positive for SARS-CoV-2 RNA. Across CT values 31–35 of the NP/OP sample, 16 (84.2 %) out of 19 saliva and one stool sample tested positive for SARS-CoV-2 RNA [Fig. 1a, b]. The correlation of CT values of NP/OP with the saliva samples was positively correlated (R-value 0.030, *p*-value 0.749), and with the stool samples was negatively correlated (R-value - 0.050, *p*-value 0. 627); however, the correlation was weak and not statistically significant [Fig. 2a, b].

**Table 1.** Characteristics of the patients with SARS-CoV2 RNA. (*n*=143)

Characteristics		*N* (%)
Age (years)	≤20	2 (1.4)
	21–59	106 (74.1)
	≥60	35 (24.5)
Sex	Male	111 (77.6)
	Female	32 (22.4)
Comorbidity	Single	52 (36.4)
	Multiple	44 (30.8)
	No comorbidities	47 (32.8)
Symptoms	Fever	124 (86.7)
	Shortness of breath, cough, chest pain	103 (72.0)
	Headache	20 (14.0)
	Loose stool, nausea, vomiting	13 (9.1)
Severity of disease	Mild	46 (32.1 %)
	Moderate	44 (30.8 %)
	Severe	53 (37.1 %)
Non-respiratory samples(*n*=345)	Saliva	116 (33.6)
	Stool	97 (28.1)
	Urine	132 (38.3)

**Table 2.** Detection of SARS-CoV-2 RNA in non-respiratory samples of patients (*n*=345)

Sample	Delectable*N* (%)	Non-detectable*N* (%)	95% Confidence Interval
Saliva (116)	102 (87.9)	14 (12.1)	93.86
Stool (*n*=97)	35 (36.1)	62 (63.9)	26.53–45.64
Urine (*n*=132)	0 (0)	132 (0)	0

**Table 3.** Prevalence of SARS-CoV2 RNA in non-respiratory samples across age, gender, comorbidity, and severity of illness (*n*=143)

Characteristics		Detectable 107 (74.8 %)	Non- detectable36 (25.2 %)	*χ²*	***p*-value**
Age	≤20 (*n*=2)	2 (100)	0 (0)	0.721	0.697
	21–59 (*n*=106)	77 (74)	29 (26)		
	≥60 (*n*=35)	28 (75.6)	7 (24.4)		
Gender	Male (*n*=111)	86 (77.4)	25 (22.6)	1.85	0.174
	Female (*n*=32)	21 (65.6)	11 (34.4)		
Comorbidity	Present (*n*=96)	75 (78.1)	21 (21.9)	1.69	0.194
	Absent (*n*=47)	32 (68.1)	15 (31.9)		
Severity of disease	Mild (*n*=46)	35 (76)	11 (24)	6.354	0.42
	Moderate (*n*=44)	38 (86.3)	6 (13.7)		
	Severe (*n*=53)	34 (64.1)	19 (35.9)		

**Table 4.** Detection of SARS-CoV2 RNA in saliva (*n*=102) and stool (*n*=35) samples across age, gender, comorbidity, and severity of illness

Characteristics		Saliva (*n*=116)102 (87.9 %)	Stool (*n*=97)35 (36.1 %)
Age	≤20 (*n*=2)	2 (100)	0 (0)
	21–59 (*n*=106)	75 (70.8)	23 (21.7)
	≥60 (*n*=35)	25 (71.4)	12 (34.3)
Gender	Male (*n*=111)	82 (73.9)	32 (28.8)
	Female (*n*=32)	20 (62.5)	3 (9.4)
Comorbidity	Present (*n*=96)	71 (74.1)	27 (28.1)
	Absent (*n*=47)	31 (66.0)	8 (17.0)
Severity of disease	Mild (*n*=46)	32 (69.6)	15 (36.2)
	Moderate (*n*=44)	38 (86.4)	13 (29.5)
	Severe (*n*=53)	32 (60.4)	7 (13.2)

## Discussion

The infectivity of COVID-19 is determined by the presence of the virus in different body fluids, secretions, and excreta. Various studies confirmed the presence of viral nucleic acid in the saliva, faeces of COVID-19 patients [15,16]. Presently, the well-recognized route of COVID-19 transmission is the inhalation of respiratory droplets in close contact. Respiratory sampling is a painful procedure and increases the risk of exposure to healthcare workers. Hence, an alternative method of sampling is today’s unmet need. The non-respiratory samples/specimens particularly self-collected could serve the purpose of an alternative method of sampling for COVID-19 diagnosis. The present study aimed to estimate the prevalence of SARS-CoV-2 in non-respiratory samples, and its correlation with demographic characteristics, comorbidities, and cycle threshold (CT) values. A total of 143 COVID-19 patients were analysed in the study and approximately 74 % of patients had ages between 21–59 years, with a mean age of 47 years. Another study by Huang C *et al*., also reported that about 49 % of cases were in the age group of 25–49 years [17]. However, Rihab *et al*. [18] observed 72 % of COVID-19 cases in the range of 21–50 years. The prevalence of COVID-19 among children is markedly low [19]. In our cohort, only two (1.39 %) cases were less than 20 years old. This age disparity could reflect the low susceptibility of children and the low propensity for symptomatic COVID-19 disease. Thus, our observation is in accordance with other published studies [19]. The majority of cases (78 %) were male, and gender distribution was also in concordance with the other studies [20,21]. However, Wang *et al*. observed an almost equal gender distribution of COVID-19 in females (51.3 %) and in males (48.7 %) [21]. Such differences in gender distribution of COVID-19 could be explained by the fact that males are more commonly involved in outdoor activity and at higher risk of acquiring infection than females or it could be a selection bias. The growing evidence suggests that COVID-19 in patients with comorbidity often developed the severe disease and required hospitalization than those without comorbidities. We noted at least one comorbidity in 67.2 % of cases. Hypertension (51), diabetes mellitus (50), and pulmonary disease (44) were the commonest comorbidities. Similarly, Wang W *et al*., and Grasselli *et al*., also observed one or more comorbidities in approximately 65–68 % of cases in their study [21,22]. However, another study showed comorbidities in only 32 % of the study population [17]. The high prevalence of comorbidity in the present study may be attributed to the selection of cases with severe diseases in patients with comorbidities requiring intensive care. It is also noteworthy, that Argenziano *et al*., also observed one or more comorbidities in 91.8 % of cases [23]. A similar spectrum of comorbidities was observed by Wang W *et al*., (hypertension 45.5 % and diabetes 22 %) and Argenziano *et al*., (hypertension 60 % and diabetes 37 %) in their cohort [21,23]. We noted underlying heart disease in 5.8 % of cases. Our observation appears to be lower than the observation by Grasseli *et al*., and such differences could be a reflection of the enrolment of the elderly (older than 80 years) patients and the high prevalence of cardiovascular disease in these elderly patients [22]. The two most common presenting symptoms in the present study were fever (86.7 %), and shortness of breath with cough and chest pain (72.0 %). Similar clinical presentations were also noted in other studies, and our findings are in concordance with the observation of such studies [24,26]. However, Yoon *et al*., did not observe fever in any case in their case series [27]. The severity of COVID-19 disease can be mild, moderate, or severe, and severity depends on the age and comorbid conditions of susceptible individuals. We noted mild disease in 46 (32 %), moderate disease in 44 (31 %), and severe disease in 53 (37 %) of COVID-19 patients. However, Guan *et al*., [28] noted severe COVID-19 disease in only 15.7 % of cases, which is very less in contrast to our observation of severe COVID-19 disease in 37 % of cases. This higher proportion of severe COVID-19 disease could be due to the high proportion of male patients, who may be more susceptible to severe disease, and the enrolment of hospitalized patients in the study. The present study was planned, with the aim to detect the SARS-CoV-2 RNA in non-respiratory samples. We analysed the real-time PCR result performed on the total 345 non-respiratory samples collected from a total of 143 real-time PCR SARS-CoV-2 RNA-positive hospitalized SARS-CoV-2 RNA in non-respiratory samples. Overall, we detected SARS-CoV-2 RNA in a total of 137 (39.7 %) non-respiratory samples. In the present study, the positivity of saliva for SARS-CoV-2 RNA was around 87.9%. Similarly, Shirazi *et al*., and Azzi *et al*., also noted a 79–100 % of positivity of saliva for SARS-CoV-2 RNA in their studies [29,30]. Another study showed 91 % prevalence in saliva whereas yet another one showed the sensitivity to be 80.7 % [31,32]. The positivity was only around 36 % in the stool sample in our study and an almost similar result was also prepared by Bwire *et al*., (32.8 %) [33]. Twenty-seven point zero three percent of stool specimens were positive for the SARS-CoV-2 genome in a study by Makhmalbaf M *et al*. [34].

However, Huang *et al*., and Shirazi *et al*., reported 68.75 and 43.7% positivity respectively which is much higher as compared to our study [17,29]. This finding may be attributed to the fact that the total number of samples was 16 as opposed to a huge number of 97 in our study. The difference in SARS-CoV-2 RNA positivity in saliva among various studies could be explained by sample collection in the morning, or avoidance of eating, drinking, and brushing teeth which may lead to a slightly higher detection rate. Another justification could be the variable dilution of saliva before processing, in different studies.

A research study by Wyllie *et al.* aiming to validate the use of saliva for SARS-CoV-2 detection found that the sensitivity of saliva SARS-CoV-2 detection is superior to nasopharyngeal swabs in early hospitalization, unlike our data [35].

We did not find SARS-CoV-2 RNA in any urine sample which is at par with the research by Pan *et al*., and Wölfel *et al*. [36,37]. Although, Huang *et al*., and Shirazi *et al*., reported SARS-CoV-2 RNA in 6.25 and 1.18 % of urine samples respectively [17,29].

When urine samples were analysed in a study by Guimarães TC *et al.* only two of the 100 patients who were admitted to the hospital tested positive for the presence of SARS-CoV-2 RNA in a urine specimen [38].

Potentially, the excretion of SARS-CoV-2 RNA in urine occurs maximally in the early stage of the disease, when patients are asymptomatic and carry a high virus load. This might be the reason behind the failure to detect SARS-CoV-2 RNA in urine samples in the present study.

We compared the overall prevalence of SARS-CoV-2 RNA in non-respiratory samples across the ages, gender, comorbidity, and severity of the disease. Prevalence of SARS-CoV-2 RNA in non-respiratory samples was higher in young (≤20 years) than in adult (21–59 years) and elderly (≥60 years) COVID-19 patients (100 % vs 74 % vs 75 %). Similarly, the prevalence of SARS-CoV-2 RNA in non-respiratory samples was higher in males than in females (77 % vs 66 %), with comorbidity than without comorbidity (78 % vs 68 %) in COVID-19 patients. The maximum (86%) prevalence of SARS-CoV-2 RNA in non-respiratory samples was among patients with moderate disease than those with mild (76 %) and severe disease (64 %). Thus, SARS-CoV-2 RNA was found in non-respiratory samples across all ages, gender, comorbidity, and severity of the disease with little difference and the differences were not statistically significant (*p*-value>0.05). We also compared the prevalence of SARS-CoV-2 RNA in saliva and stool individually across the ages, gender, comorbidity, and severity of the disease. The prevalence of SARS-CoV-2 RNA in saliva was 70–100 % across three groups of age, while it was only 0–34 % in the stool sample. Similarly, the prevalence of SARS-CoV-2 RNA was higher in males than in females in saliva (74 % vs 62 %) as well as in stool (29 % vs 9 %) samples. A high prevalence of SARS-CoV-2 RNA was noted in patients with comorbidity than without, in saliva (74 % vs 66 %) as well as in stool (28 % vs 17 %) samples. The maximum (86%) prevalence of SARS-CoV-2 RNA in saliva was among patients with moderate disease than those with mild (70 %) and severe disease (60 %); while in stool samples it was maximum (36%) in patients with mild disease. Although, SARS-CoV-2 RNA was detected in saliva and stool samples across all ages, gender, comorbidity, and severity of the disease; the difference in positivity between the two samples could be explained by a greater number of saliva samples analysed, low viral load in stool or technical issues in real-time PCR tests.

The CT value of the ORF gene in 102 saliva and 35 stool samples were grouped and designated as high (CT value <25), medium (CT value 25–30), and low (CT value >30) viral load samples respectively. Approximately half (52%) of the saliva sample had a high viral load, the rest of the 34 % sample had medium and 14 % had a low viral load. In contrast, Bhatta A *et al*. [39] reported high viral load in only 21.5 % of saliva samples. The majority (78 %) of stool samples had a low viral load and the rest 22 % had a medium viral load. None of the stool samples had a high viral load. In a study, Cerrada-Romero *et al*., [40] noted CT values of the positive stool samples between 24.5 to 39.6; which corresponds to the medium to low viral load of our observation. While respiratory fluids have a higher shedding prevalence than faeces, the study model by Crank K *et al.* is consistent with stool RNA dominating in wastewater for community shedding [41].

We performed Pearson correlation analysis; between CT values of the ORF 1ab gene in nasopharyngeal/ oropharyngeal (NP/OP) and saliva samples, as well as between CT values ORF 1ab gene in NP/OP and stool samples. The correlation between CT values of NP/OP and saliva is positively correlated (R-value 0.030), and between CT values of NP/OP and stool is negatively correlated (R-value −0.050); however, the correlation is weak and statistically not significant (*p*-value>0.05). However, a statistically significant correlation between the CT value of NS/OP and saliva was observed in another study done by Genelhoud G *et al*., (*P*=0.002) [42].

## Conclusion

We could demonstrate the presence of SARS-CoV-2 RNA in 107 (74.8 %) patients, in at least one of the non-respiratory samples. The viral RNA was detected in 137 (40 %) non-respiratory (102 [88 %] saliva and 35 [36 %] stool) samples. The SARS-CoV-2 RNA was undetectable in any of the urine samples. Approximately half (52 %) of the saliva samples had a high viral load while majority (78%) of stool samples had a low viral load. Hence, the non-respiratory samples especially the saliva may be an alternative self-collected specimen for first-line screening test for COVID-19 diagnosis. This might help to decrease the risk of exposure to healthcare workers while collecting respiratory samples.

The presence of SARS-CoV-2 RNA in the faeces of COVID-19 patients indicates potential feco-oral transmission of SARS-CoV-2. In addition to this, the excretion of SARS-CoV-2 in human stool and saliva is a concern of public health importance, especially in developing countries like India where open defecation and spitting in public is a rampant practice. Also, stray animals may get infected and might serve as a source of infection for humans. The sewage system might also be a potential source of viral outbreaks, community spread, and a future surveillance strategy. However, these potential routes of transmission need further investigation.

This study signifies the potential role of non-respiratory samples in environmental surveillance. A well-planned sewage surveillance during the suspected season for COVID-19 may provide an early signal for a potential outbreak situation.

The transmission to the sewage system, poor sanitation, and insufficient treatment in poorer countries can lead to non-point contamination of surface waters.

## Limitations

- The follow-up of the patients and collection of multiple serial non-respiratory samples could have been done to know the exact time to clearance of the SARS-CoV-2 RNA from the body fluids.
- This study is a single-centre study. Additionally, the study was done on positive cases only which can’t be generalized to a normal population.
- The samples were collected only from the admitted cases hence the excretion of the SARS CoV-2 RNA in the non-respiratory samples of the mild cases who were in home isolation may not be told.

## Abbreviations

COVID-19: coronavirus disease 2019
CT: cycle threshold
IQR: inter quartile range
NP/OP: nasopharyngeal/oropharyngeal
PBS: phosphate buffered saline
RNA: ribonucleic acid
RT-PCR: real-time polymerase chain reaction
SARS-CoV-2: severe acute respiratory syndrome coronavirus 2
SD: standard deviation
VRDL: Virus Research and Diagnostic Laboratory
VTM: viral transport media
